# Identification of Plant Quantitative Trait Loci Modulating a Rhizobacteria-Aphid Indirect Effect

**DOI:** 10.1371/journal.pone.0041524

**Published:** 2012-07-26

**Authors:** Catherine Tétard-Jones, Michael A. Kertesz, Richard F. Preziosi

**Affiliations:** Faculty of Life Sciences, The University of Manchester, Manchester, United Kingdom; Pennsylvania State University, United States of America

## Abstract

Plants simultaneously interact with a plethora of species both belowground and aboveground, which can result in indirect effects mediated by plants. Studies incorporating plant genetic variation indicate that indirect effects mediated by plants may be a significant factor influencing the ecology and evolution of species within a community. Here, we present findings of a Quantitative Trait Locus (QTL) mapping study, where we mapped a rhizobacteria-aphid indirect effect onto the barley genome. We measured the size of aphid populations on barley when the barley rhizosphere either was or was not supplemented with a rhizobacterial species. Using a QTL mapping subset, we located five regions of the barley genome associated with the rhizobacteria-aphid indirect effect. Rhizobacterial supplementation led to an increase in aphid population size (mapped to three barley QTL), or a decrease in aphid population size (mapped to two barley QTL). One QTL associated with plant resistance to aphids was affected by a significant QTL-by-environment interaction, because it was not expressed when rhizobacteria was supplemented. Our results indicated that rhizobacterial supplementation of barley roots led to either increased or reduced aphid population size depending on plant genotype at five barley QTL. This indicates that the direction of a rhizobacteria-aphid indirect effect could influence the selection pressure on plants, when considering species that affect plant fitness. Further research may build on the findings presented here, to identify genes within QTL regions that are involved in the indirect interaction.

## Introduction

As sessile organisms, plants simultaneously interact with and produce responses to a multitude of interacting species both belowground and aboveground. Although mostly studied in separation, the ecology of belowground and aboveground communities is connected via induced plant responses [1], [2], [3], [4]. It is increasingly recognised that the ecology and evolution of species within a community are strongly interdependent and this has been the subject of an upsurge in studies of eco-evolutionary dynamics (the evolution of multiple interacting species’ in response to their reciprocal interactions within a community) and community genetics [5], [6], [7], [8], [9], [10], [11]. Chains of directly interacting and co-evolving species can lead to indirect interactions at further trophic levels, such as rhizosphere bacteria (rhizobacteria)-plant-insect herbivore interactions. Indirect interactions may have a significant impact on the eco-evolutionary dynamics of communities [12], particularly when they are stronger than or reverse the direction of the direct effects [13] via induced plant responses [5]. The strength of indirect interactions can influence the selection of plant induced responses that maximise indirect interactions when an indirect effect results in enhanced plant fitness, as demonstrated by plants’ evolved ability to attract insect predators via plant volatiles [14], [15], [16]. The ability for indirect effects to reverse the direction of direct effects can be seen in studies of pathogenic or plant growth promoting rhizobacteria and mycorrhizal fungi that enhance plant resistance to further diseases or insect pests [2], [17], [18], [19], [20], [21]. Rhizobacterial induced plant defences to pests and disease present an example of diffuse evolution whereby a selection pressure or the response to selection imposed by one species on another may depend on the presence or absence of other species within the community [22].

Whether a selection pressure caused by indirect effects results in an altered evolutionary trajectory of plant responses depends on whether intraspecific genetic variation associated with those responses influences the outcome of the indirect effect on plant fitness. Intraspecific genetic variation can influence the outcome of indirect effects by affecting the transmission of the indirect effect by the sender species [23], [24], mediation of the indirect effect by the mediator species [20], [25], and how the indirect effect is received [20], [26]. In a recent study, supplementation of the rhizobacterial community with a single rhizobacterial species was shown to influence aphid fitness either positively (increased population size) or negatively (decreased population size) [20] depending on the combination of plant genotype and aphid genotype. This study provides a basis for focusing in on the underlying mechanisms that are responsible for variation in indirect effects by using Quantitative Trait Locus (QTL) mapping. QTL mapping is a technique for locating regions of the genome that are associated with quantitative traits, such as induced plant responses. The technique works by testing whether genetic variation at loci is responsible for a significant difference in the measured trait. Thus it can be used to map the effects of genetic variation on the direction or strength of direct and indirect effects to specific regions of a chromosome. Locating QTL can aid the identification of the individual genes within QTL regions that are involved. Although QTL mapping of direct effects has been extensively studied [e.g. 27,28,29,30,31,32], QTL mapping of indirect effects is rarely conducted, and has the potential to contribute to our understanding of the mechanisms underlying the ecology and evolution of species [33], [34].

In a recent study, we [35] used contrasting rhizosphere treatments to map plant QTL and QTL-by-environment interactions associated with phenotypic plasticity in barley-aphid interactions. This study demonstrated that a small subset of a QTL mapping population (consisting of 50 lines) can be used to locate multiple QTL associated with multi-trophic interactions when logistical constraints prevent the use of the full mapping population. The use of a small mapping population is known to cause a reduced ability to detect small effect QTL (resulting in fewer significant QTL) and overestimation of QTL effects compared to mapping with the full QTL population [36]. Despite the latter problem, mapping with a subset of the full population has not been shown to affect the likelihood of detecting false positives.

In the current study, we used a rhizobacteria-barley-aphid model ecosystem to map a belowground-aboveground indirect effect onto the barley genome. Our aims were to: 1) quantify the indirect effect of rhizosphere supplementation with a rhizobacterial species (*Pseudomonas aeruginosa* 7NSK2) on aphid population size across Doubled Haploid (DH) lines of a barley Quantitative Trait Locus (QTL) mapping population; 2) locate barley QTL associated with the rhizobacteria-aphid indirect effect, in order to find regions of the barley genome that are associated with a change in plant response/resistance to aphids under contrasting rhizobacterial environments. We discuss how our results indicate a potential mechanism for the rhizobacteria-aphid indirect effect, and how such a mechanism could influence eco-evolutionary dynamics of plant-insect interactions. The QTL regions located in this study could provide a basis for future studies that seek to identify genes involved in the rhizobacteria-aphid indirect effect.

## Materials and Methods

### Experimental Design

We mapped barley (*Hordeum vulgare*) QTL associated with the population size of the cereal aphid (*Sitobion avenae* clone HF92a, previously described in Tétard-Jones *et al* [20]) using a doubled haploid (DH) barley mapping population derived from Oregon Wolfe Barley Dominant and Recessive (OWB) parental lines. DH populations are used in many cereal crops for mapping QTL, due to the homozygous lines produced using the bulbosum technique [37]. The OWB population has a high average mapped marker density (5.5cM) over seven chromosomes; Ch1: 136cM, 29 markers; Ch2: 180cM, 35 markers; Ch3: 218cM, 28 markers; Ch4: 125cM, 31 markers; Ch5: 225cM, 37 markers; Ch6: 167cM: 35 markers; Ch7: 199cM, 37 markers. The linkage map for the ninety four line population is available on the GrainGenes website: http://www.wheat.pw.usda.gov/GG2/index.shtml. Seeds for the mapping population were supplied by P. Hayes (Oregon State University). In this study, a subset consisting of fifty lines were selected at random from the OWB population for phenotyping and subsequent mapping. Two environments were set up: 1) rhizosphere supplementation with *Pseudomonas aeruginosa* 7NSK2; 2) control (no rhizosphere supplementation). Each of the 50 DH and 2 parental lines were treated in each environment, and each line-environment combination was replicated four times, producing 416 plants. We used a randomized block design with replicate used as the block and each line-environment combination was randomized within each replicate block. Although this experimental design is presented as model system rather than a field trial, it is likely that the species used interact with each other in naturally occurring communities. The rhizobacterial strain *P. aeruginosa* 7NSK2 is an isolate from barley roots (Iswandi *et al.,* 1987) and the aphid *Sitobion avenae* (English grain aphid) is a herbivore of all grass species.

### Plant Growth and Phenotyping

We phenotyped the lines for aphid fitness in a glasshouse at the Firs Experimental Research Station (The University of Manchester) during June 2006. Supplemental lights were used to provide a 16∶8 light:dark regime and a daily temperature range of 16–30°C. Seeds were sterilized with 10% sodium hypochlorite (followed by several washes with sterilized water) and germinated in sterile Petri dishes and filter paper for five days. Preparation of the *P. aeruginosa* inoculum and seedling inoculation were performed as previously described [20]. After inoculation, seedlings were planted into 10 cm pots containing heat sterilized horticultural grade sharp sand. Plants were watered twice daily via their saucers, and fed once a week with 40 ml full strength Hoagland’s solution [38]. Eleven days after transplanting the seedlings, two adult aphids were placed onto each plant, and each plant was enclosed in a transparent tube with mesh windows. Two weeks after aphid infestation, the resulting aphid population size for each plant was counted as our measure of aphid fitness. Plants were collected, washed to remove sand and cut into root and shoot sections. Root and shoot samples were oven dried for 3 days at 80°C, for dry biomass assessment.

### Data Analysis

QTL analysis was conducted with the means of the aphid measurements, calculated from four plant replicates. We used the Composite Interval Mapping (CIM) procedure in windows QTLcartographer v2.5 [39]. This method tests the association between trait values and genotype values (actual genotype values at marker sites and inferred genotype values modelled by QTLcartographer at 2cM intervals along each chromosome). At each marker site and 2cM interval, the QTL analysis includes background markers as cofactors to control for variance caused by QTL at non target loci outside the flanking markers, as determined by the window size. We used a window size of 10cM. Values for r^2^ (percentage phenotypic variation explained by a QTL) and additive genetic effect were generated by windows QTLcartographer. The location of a QTL on each chromosome was defined as the point where the Log of the Odds ratio (LOD, provides a measure of the association between variation in a measured trait and genetic differences (alleles) at a loci) exceeded the threshold value. Threshold values were calculated for each chromosome following the chromosome-wise approach of Li and Ji [40]. This method involves calculation of the effective number of marker loci using results from Principal Components Analysis of the marker data. Chromosome-wise threshold levels can be less conservative than genome-wise threshold values, and increase discovery of true positives whilst avoiding problems using the false discovery rate [41], [42]. For comparison, genome-wise threshold levels calculated in windows QTLcartographer using 1000 permutations at p<0.05 gave LOD thresholds of 2.98 and 2.50 for the non-supplemented and supplemented rhizosphere environments respectively. QTL-by-Environment interactions (QTLxE) were tested using PROC GLM in SAS.

## Results

### Phenotypic Effect of Pseudomonas Aeruginosa 7NSK2 Supplementation-barley Interaction on Aphid Fitness

**Figure 1:**
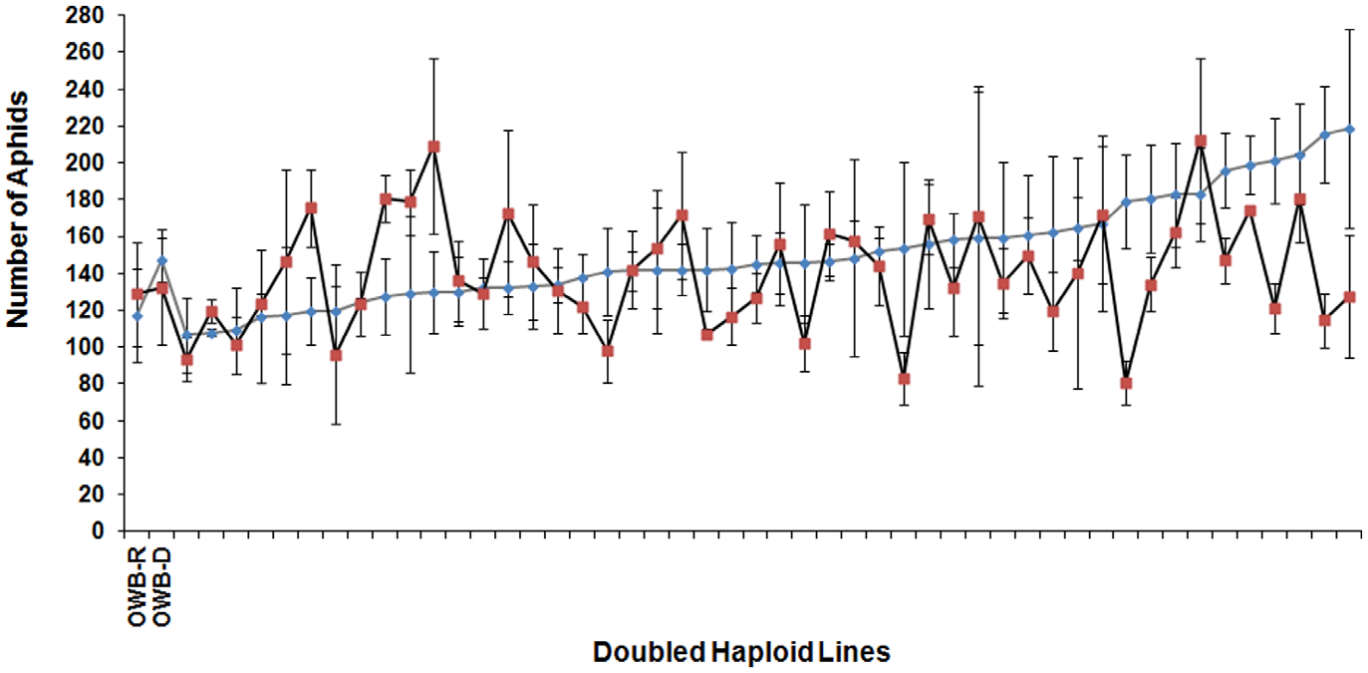
Variation in aphid fitness (number of aphids) among barley mapping lines. The mapping lines are arranged in ascending order according to the number of aphids on mapping lines when *Pseudomonas aeruginosa* 7NSK2 had not been supplemented (diamonds connected by a grey line). The squares connected with a black line shows the number of aphids on mapping lines when *Pseudomonas aeruginosa* 7NSK2 was supplemented. Parental lines (OWB-D and OWB-R) are also shown for reference.

Rhizosphere supplementation with *P. aeruginosa* 7NSK2 led to an increased aphid population size on 51% of 48 barley genotypes and reduced aphid population size on 36% of 48 barley mapping population lines (Fig. 1) compared to the control (no rhizosphere supplementation). The data for two mapping lines (line ID 38 and 57) was excluded due to inconsistency in the infestation across all replicates of those lines.

### Pseudomonas Aeruginosa 7NSK2 Supplementation Modifies the Effect of Plant QTL on Aphid Population Size

**Figure 2:**
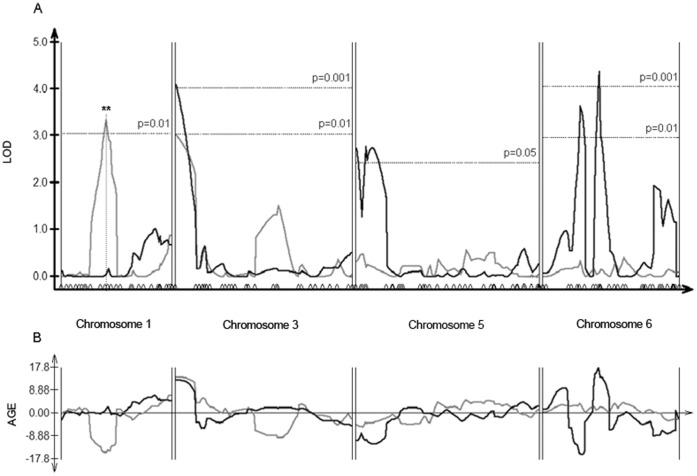
Association between barley loci and aphid fitness. A) QTL plot and B) additive genetic effect plot, showing the association between barley loci and aphid fitness along chromosomes 1, 3, 5 and 6. In figure A, the lines show the level of association (LOD, y-axis) between barley loci and aphid fitness at each marker position (shown by small peaks along the x-axis) when the barley rhizosphere was supplemented with *Pseudomonas aeruginosa* 7NSK2 (black line) and when the barley rhizosphere was not supplemented (grey line). The dotted horizontal lines extending across the plots show the chromosome-wise threshold levels required for a barley loci-aphid association to be a significant QTL (p values indicated for each threshold line). Asterisk show the location of significant QTLxE, ** = p<0.01. In figure B, the AGE (additive genetic effect) indicates the parental allele that contributed to the QTL (positive AGE  =  OWB-D, negative AGE  =  OWB-R) and the magnitude of effect of the allele at each marker position for each environment (rhizobacteria supplemented vs. non-supplemented).

Barley QTL were mapped for their association with aphid fitness (population size) in both environmental data sets (i.e. with and without *P. aeruginosa* 7NSK2 supplementation). We located five QTL, on chromosomes 1, 3, 5 and 6 using chromosome-wise threshold levels (Table 1, Fig. 2a). Under genome-wise threshold levels (LOD 2.98 and 2.50 for the non-supplemented and supplemented rhizobacterial environments respectively), the same set of QTLs are significant (p<0.05). Significant QTL explained between 10–20% of phenotypic variation. One QTL was significant in both environments (chromosome 3, Fig. 2a), though at a higher level of significance when the barley rhizosphere was supplemented with *P. aeruginosa* 7NSK2. For the QTL on chromosomes 5 and 6, *P. aeruginosa* 7NSK2 supplementation caused an increased association between the barley QTL and aphid fitness, which resulted in the significant QTL. In contrast, on chromosome 1, *P. aeruginosa* 7NSK2 supplementation reduced the association between QTL and aphid fitness, which resulted in a significant QTL-by-environment interaction (QTLxE). Additive Genetic Effect (AGE) values indicated that QTL associated with positive/negative effects on aphids could be contributed by alleles of either parent (Fig. 2b). For example, on chromosome 1 the QTL was associated with reduced aphid fitness (parent OWB-D contributing negative effect alleles) when *P. aeruginosa* 7NSK2 was not supplemented. On chromosome 3, the QTL was associated with increased aphid fitness (parent OWB-D contributing positive effect alleles) in both environments. This demonstrates that multiple alleles from the same parental genotype can have opposing effects on the induced plant response to aphids. Furthermore, the direction and magnitude of this effect is influenced by the supplementation of the rhizosphere with *P. aeruginosa* 7NSK2.

**Table 1:** Positions and effects of QTL located on chromosomes one, three, five and six.

QTL position^1^	Rhizobacteria	LOD	Phenotypic variation explained by QTL [%]	Additive genetic effect^2^	Indirect effect with OWB-D^3^
Ch1, 54.11cM	*Supplemented	0.05	0.15	1.32	
	*Not supplemented	3.34	17.78	−14.93	Fewer aphids
Ch3, 0.00cM	Supplemented	4.10	17.90	12.97	More aphids
	Not supplemented	3.01	15.82	14.02	More aphids
Ch5, 11.35cM	Supplemented	2.78	10.87	−10.21	Fewer aphids
	Not supplemented	0.21	0.88	−3.18	
Ch6, 44.85cM	Supplemented	3.64	15.29	−15.15	Fewer aphids
	Not Supplemented	0.32	1.50	3.92	
Ch6, 68.00cM	Supplemented	4.38	19.13	17.76	More aphids
	Not Supplemented	0.10	0.44	2.15	

Notes:

1 The position of the nearest marker to the maximum LOD with the QTL region.

2 The additive genetic effect reflects a) the magnitude of the allele effect on aphid fitness at the point of maximum LOD, b) which parental allele produced the significant QTL, positive AGE  =  the OWB-D and negative AGE  =  OWB-R.

3 The consequence of the OWB-D allele on the rhizobacteria-aphid indirect effect compared to the OWB-R allele in the environment where the QTL was significant.

* QTLxE on chromosome 1, p = 0.0018.

From a perspective of the rhizobacterial induced plant response, the indirect effect of *P. aeruginosa* 7NSK2 supplementation on aphid fitness was influenced by plant QTL. In two cases, *P. aeruginosa* 7NSK2 supplementation led to an increase in aphid population size. This was due in the first case to the suppression of the negative plant response QTL that was located in the control environment (chromosome 1), and in the second case to enhancement of the positive effect QTL located in the supplemented environment (chromosome 6, 68cM). The negative effect of *P. aeruginosa* 7NSK2 supplementation was associated with the QTL at Ch6, 45cM.

### Differential Effects of Rhizobacterial Supplementation on Plant and Aphid Performance

**Figure 3:**
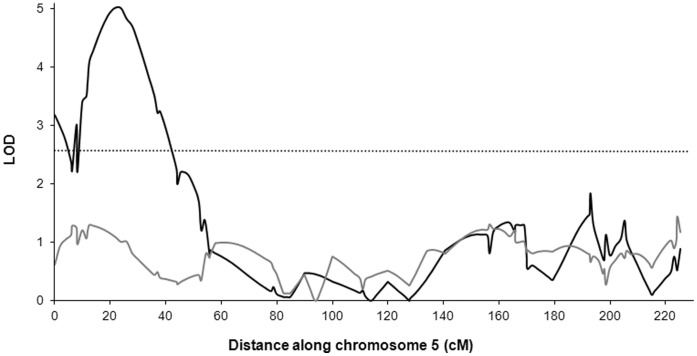
Association between barley loci and aphid fitness when plant shoot biomass was a covariate. The QTL plot shows the level of association (LOD, y-axis) between barley loci and aphid fitness along chromosome 5 (x-axis) when barley shoot biomass was used as a covariate. The lines show the association of aphid fitness with (black) and without (grey) rhizosphere supplementation with *Pseudomonas aeruginosa* 7NSK2. The dotted horizontal line shows the chromosome-wise threshold for a significant QTL (p<0.05). The increased association between barley loci and aphid fitness when shoot biomass was used as a covariate compared to not using a covariate (shown in figure 2) indicates that the effect of rhizobacteria on aphid fitness was not explained by a plant growth effect of the rhizobacteria.

*Pseudomonas aeruginosa* 7NSK2 is reported to stimulate plant growth [43]. We therefore tested whether the effect of *P. aeruginosa* 7NSK2 supplementation was due to the effect of the inoculant on plant growth. If this were true, *P. aeruginosa* 7NSK2 supplementation could have made these plants better hosts for aphids by increasing the physical size of the plants. We used dry shoot biomass as a covariate to test whether it would alter the mapping of QTL associated with aphid fitness. The covariate had the largest effect on the QTL on chromosome 5 (Fig. 3). When we mapped with the covariate, the association of the QTL with aphid fitness increased, suggesting that shoot biomass was associated with a negative rather than positive influence on aphid fitness. This QTL also corresponded to a QTL for increased shoot biomass in a previous study. The QTL had opposing effects on each species trait; therefore the alleles of parent OWB-D had a negative effect on aphid fitness and a positive effect on shoot biomass. The QTL that influenced aphid fitness and shoot biomass were significant only when the rhizosphere was supplemented with *P. aeruginosa* 7NSK2. This suggests that the QTL modulates the plant induced response when *P. aeruginosa* 7NSK2 is present.

## Discussion

Using a model tritrophic ecosystem, we have demonstrated that a belowground-aboveground indirect effect of rhizobacteria on aphid fitness was mediated by barley genetic variation at five barley QTL. The QTL displayed opposing effects on aphid fitness, in agreement with previous work on rhizobacteria-barley-aphid interactions, in that rhizobacteria can have either positive or negative effects on aphids depending on plant genotype [20]. Here we have shown that those opposing indirect effects were mediated by multiple barley QTL.

### Plant QTL Link the Rhizobacteria-aphid Indirect Effect

For two barley QTL, *P. aeruginosa* 7NSK2 supplementation had a positive impact on aphid fitness (QTL on chromosomes 3 and 6). This effect may be explained by increased plant host quality. Rhizobacteria can modify plant hormone status, leading to increased nutrient or water uptake by plants and changes in transpiration rate and phloem sap composition [44], [45], [46]. Alterations in phloem sap composition modifies plant host quality for sap feeders such as aphids [44], [47] and is directly linked to aphid fitness. The QTL on chromosome 1 also demonstrated a positive effect of *P. aeruginosa* 7NSK2 supplementation, due to a suppression of the barley response to aphids that occurred in the control environment. The QTLxE interaction indicated the location of genes that were differentially expressed in contrasting rhizosphere environments, which resulted in phenotypic plasticity of resistance to aphids [35]. Two barley QTL linked *P. aeruginosa* 7NSK2 supplementation to a decrease in aphid population size (chromosomes 5 and 6). Rhizobacterial inoculation of plant roots with *P. aeruginosa* 7NSK2 can lead to an Induced Systemic Resistance [ISR, 2]. Although ISR primarily acts as a plant defence against foliar pathogens, a rhizobacterial ISR could explain the enhanced plant resistance of some barley lines to aphids, since there is some cross-over in plant responses to pathogen and insect pests [48]. The QTL found in our study co-locate to several genes involved in plant-pathogen interactions. Marker ABG395 (flanking the QTL on chromosome 5) is mapped nearby the gene for a lipid transfer protein (Ltp1), which has a role in powdery mildew resistance in several barley and wheat mapping populations [49]. This marker is also linked to a QTL affecting plant response to the fungal disease net blotch in the Steptoe × Morex barley mapping population [50]. Marker BCD907 (chromosome 3) is linked to a gene associated with leaf rust resistance (Rph6) [51]. Further investigation could confirm the expression of defence related genes at QTL positions (position of maximum LOD in Table 1) within the QTL regions mapped in this study.

Rhizobacterial ISR functions by priming plant genes to plant pathogens, so that these genes respond faster or more strongly upon pathogen attack [2], [3]. A major difference between our study and those investigating Rhizobacteria ISR is that in our system the rhizosphere treatment was a supplementation of a single rhizobacterial species (*P. aeruginosa* 7NSK2), rather than the absolute presence/absence of rhizobacteria. In our study the plant seeds and growing medium (sand) were sterilised prior to rhizosphere supplementation with *P. aeruginosa* 7NSK2. Following transplantation the rhizosphere was allowed to be colonised by naturally occurring micro-organisms, which may have entered the system for example via the irrigation system. Therefore, the contrasting direction of QTL effects on aphid fitness in our study may be attributed to the supplementation of *P. aeruginosa* 7NSK2 within a rhizobacterial community, and possibly a more complex indirect effect arising from the altered rhizobacterial community composition rather than an indirect effect from a single rhizobacterial strain.

### Plant QTL Associated with Rhizobacteria-aphid Indirect Effect: A Mechanism of Eco-evolution?

It is well established that plant stress from herbivorous insects exerts a selection pressure on plants to evolve traits that maximise their defence due to the fitness cost of insect attack. For selection to result in evolution, genetic variation is needed at plant loci that alter plant resistance. In this study, we have mapped barley QTL that alter plant resistance to aphids with rhizobacterial supplementation. These results indicate a potential for selection to act on plant genotypes that have an altered insect resistance depending on the composition of the rhizobacterial community, which in turn could alter the long term dynamics of insect populations [52]. This study indicates that the direction of the selection pressure would depend on the genotype at multiple QTL. Whether this would contribute to eco-evolutionary dynamics, in which the ecology and evolution of belowground and aboveground plant communities can be interdependent, may ultimately depend on eco-evolutionary feedback.

### The Consequences of Eco-evolutionary Feedback Mechanisms for the Ecology and Evolution of Plant-insect Interactions

The driver of antagonistic plant-insect eco-evolutionary dynamics is that selection resulting in a fitness advantage for one species results in a lower fitness and hence a greater selection pressure on the interacting species. Hence a fitness advantage that plants obtain from rhizobacterial induced plant defence could feedback into a greater selection pressure on insects to counteract the indirect effect of rhizobacteria. Plant induced responses can mediate indirect effects from the belowground-aboveground for example via altered plant nutrient levels, plant volatile signals received by parasitoids, and induced defence [15], [44], [53], [54], [55]. A recent study indicates that plant mediated indirect effects can influence herbivore trait evolution [1]. This could lead to the induction of aboveground-belowground indirect effects [4] via plant induced responses. For example foliar herbivory can lead to increased carbon release from plant roots, which promotes microbial activity and increases nitrogen availability [56], and provides greater nutrition for foliar herbivores. Plant induced responses to insects may also lead to the selection of ‘coping mechanisms’, whereby insects detoxify plant defensive compounds in the insect gut [57], [58], [59]. The antagonistic arms race between plants and insects suggests that an advantage to the plant due to other interacting species such as rhizobacteria could lead to feedback from insect herbivores.

## References

[1] Bonte D, De Roissart A, Vandegehuchte ML, Ballhorn DJ, Van Leeuwen T, et al (2010) Local adaptation of aboveground herbivores towards plant phenotypes induced by soil biota. PLoS One 5: 9. 10.1371/journal.pone.0011174 PMC2887358 20567507

[2] van Loon LC, Bakker PAHM, Pieterse CMJ (1998) Systemic resistance induced by rhizosphere bacteria. Annu Rev Phytopathol 36: 453–483. 15012509 10.1146/annurev.phyto.36.1.453

[3] Verhagen BWM, Glazebrook J, Zhu T, Chang HS, van Loon LC, et al (2004) The transcriptome of rhizobacteria-induced systemic resistance in Arabidopsis. Mol Plant-Microbe Interact 17: 895–908. 15305611 10.1094/MPMI.2004.17.8.895

[4] Wardle DA, Bardgett RD, Klironomos JN, Setala H, van der Putten WH, et al (2004) Ecological linkages between aboveground and belowground biota. Science 304: 1629–1633. 15192218 10.1126/science.1094875

[5] Utsumi S (2011) Eco-evolutionary dynamics in herbivorous insect communities mediated by induced plant reponses. Popul Ecol 53: 23–34.

[6] Pelletier F, Garant D, Hendry AP (2009) Eco-evolutionary dynamics. Phil Trans R Soc B 364: 1483–1489. 19414463 10.1098/rstb.2009.0027 PMC2690510

[7] Bailey JK, Hendry AP, Kinnison MT, Post DM, Palkovacs EP, et al (2009) From genes to ecosystems: an emerging synthesis of eco-evolutionary dynamics. New Phytol 184: 746–749. 20021593 10.1111/j.1469-8137.2009.03081.x

[8] Wade MJ (2007) The co-evolutionary genetics of ecological communities. Nat Rev Genet 8: 185–195. 17279094 10.1038/nrg2031

[9] Hersch-Green EI, Turley NE, Johnson MTJ (2011) Community genetics: what have we accomplished and where should we be going? Phil Trans R Soc B 366: 1453–1460. 21444318 10.1098/rstb.2010.0331 PMC3081577

[10] Whitham TG, Bailey JK, Schweitzer JA, Shuster SM, Bangert RK, et al (2006) A framework for community and ecosystem genetics: from genes to ecosystems. Nat Rev Genet 7: 510–523. 16778835 10.1038/nrg1877

[11] Johnson MTJ, Stinchcombe JR (2007) An emerging synthesis between community ecology and evolutionary biology. Trends Ecol Evol 22: 250–257. 17296244 10.1016/j.tree.2007.01.014

[12] Wootton JT (1994) The nature and consequences of indirect effects in ecological communities. Annu Rev Ecol Syst 25: 443–466.

[13] Miller TE, Travis J (1996) The evolutionary role of indirect effects in communities. Ecology 77: 1329–1335.

[14] Snoeren TAL, De Jong PW, Dicke M (2007) Ecogenomic approach to the role of herbivore-induced plant volatiles in community ecology. J Ecol 95: 17–26.

[15] Soler R, Harvey JA, Kamp AFD, Vet LEM, Van der Putten WH, et al (2007) Root herbivores influence the behaviour of an aboveground parasitoid through changes in plant-volatile signals. Oikos 116: 367–376.

[16] van Loon JJA, de Boer JG, Dicke M (2000) Parasitoid-plant mutualism: parasitoid attack of herbivore increases plant reproduction. Entomol Exp Appl 97: 219–227.

[17] Goellner K, Conrath U (2008) Priming: it’s all the world to induced disease resistance. Eur J Plant Pathol 121: 233–242.

[18] Stout MJ, Zehnder GW, Baur ME (2002) Potential for the use of elicitors of plant resistance in arthropod management programs. Arch Insect Biochem Physiol 51: 222–235. 12432521 10.1002/arch.10066

[19] Bennett AE, Alers-Garcia J, Bever JD (2006) Three-way interactions among mutualistic mycorrhizal fungi, plants, and plant enemies: hypotheses and synthesis. Am Nat 167: 141–152. 16670976 10.1086/499379

[20] Tétard-Jones C, Kertesz MA, Gallois P, Preziosi RF (2007) Genotype-by-genotype interactions modified by a third species in a plant-insect system. Am Nat 170: 492–499. 17879200 10.1086/520115

[21] Zytynska SE, Fleming S, Tetard-Jones C, Kertesz MA, Preziosi RF (2010) Community genetic interactions mediate indirect ecological effects between a parasitoid wasp and rhizobacteria. Ecology 91: 1563–1568. 20583697 10.1890/09-2070.1

[22] Strauss SY, Sahli H, Conner JK (2005) Toward a more trait-centered approach to diffuse (co)evolution. New Phytol 165: 81–90. 15720623 10.1111/j.1469-8137.2004.01228.x

[23] Bailey JK, Wooley SC, Lindroth RL, Whitham TG (2006) Importance of species interactions to community heritability: a genetic basis to trophic-level interactions. Ecol Lett 9: 78–85. 16958871 10.1111/j.1461-0248.2005.00844.x

[24] Fuentes-Contreras E, Niemeyer HM (2002) Direct and indirect effects of wheat cultivars with different levels of resistance on parasitoids and entomopathogenic fungi of cereal aphids. Ecoscience 9: 37–43.

[25] Cronin JT, Abrahamson WG (2001) Goldenrod stem galler preference and performance: effects of multiple herbivores and plant genotypes. Oecologia 127: 87–96. 28547173 10.1007/s004420000561

[26] Astles PA, Moore AJ, Preziosi RF (2005) Genetic variation in response to an indirect ecological effect. Proc R Soc Biol Sci Ser B 272: 2577–2581. 10.1098/rspb.2005.3174 PMC1559979 16321778

[27] Via S, Hawthorne DJ (2002) The genetic architecture of ecological specialization: Correlated gene effects on host use and habitat choice in pea aphids. Am Nat 159: S76–S88. 18707371 10.1086/338374

[28] Alam SN, Cohen MB (1998) Detection and analysis of QTLs for resistance to the brown planthopper, *Nilaparvata lugens*, in a doubled-haploid rice population. Theor Appl Genet 97: 1370–1379.

[29] Bonierbale MW, Plaisted RL, Pineda O, Tanksley SD (1994) QTL analysis of trichome-mediated insect resistance in potato. Theor Appl Genet 87: 973–987. 24190532 10.1007/BF00225792

[30] Castro AM, Vasicek A, Ellerbrook C, Gimenez DO, Tocho E, et al (2004) Mapping quantitative trait loci in wheat for resistance against greenbug and Russian wheat aphid. Plant Breed 123: 361–365.

[31] Moharramipour S, Tsumuki H, Sato K, Yoshida H (1997) Mapping resistance to cereal aphids in barley. Theor Appl Genet 94: 592–596.

[32] Soundararajan RP, Kadirvel P, Gunathilagaraj K, Maheswaran M (2004) Mapping of quantitative trait loci associated with resistance to brown planthopper in rice by means of a doubled haploid population. Crop Sci 44: 2214–2220.

[33] Erickson DL, Fenster CB, Stenoien HK, Price D (2004) Quantitative trait locus analyses and the study of evolutionary process. Mol Ecol 13: 2505–2522. 15315666 10.1111/j.1365-294X.2004.02254.x

[34] Smith KP, Handelsman J, Goodman RM (1999) Genetic basis in plants for interactions with disease-suppressive bacteria. Proc Natl Acad Sci U S A 96: 4786–4790. 10220371 10.1073/pnas.96.9.4786 PMC21769

[35] Tétard-Jones C, Kertesz MA, Preziosi RF (2011) Quantitative trait loci mapping of phenotypic plasticity and genotype-environment interactions in plant and insect performance. Phil Trans R Soc B 366: 1368–1379. 21444311 10.1098/rstb.2010.0356 PMC3081578

[36] Vales MI, Schon CC, Capettini F, Chen XM, Corey AE, et al (2005) Effect of population size on the estimation of QTL: a test using resistance to barley stripe rust. Theor Appl Genet 111: 1260–1270. 16179997 10.1007/s00122-005-0043-y

[37] Jensen CJ (1976) Barley monoploids and doubled monoploids: techniques and experience. Barley Genetics III: Proceedings of the 3rd International barley genetics symposium. 316–345.

[38] Hoagland DR, Arnon DI (1950) The water-culture method for growing plants without soil. California Agricultural Experiment Station Circular 347: 1–32.

[39] Basten CJ, Weir BS, Zeng Z-B (2002) QTL Cartographer. 1.16 ed. Raleigh, NC: Department of Statistics, North Carolina State University.

[40] Li J, Ji L (2005) Adjusting multiple testing in multilocus analyses using the eigenvalues of a correlation matrix. Heredity 95: 221–227. 16077740 10.1038/sj.hdy.6800717

[41] Chen L, Storey JD (2006) Relaxed Significance Criteria for Linkage Analysis. Genetics 173: 2371–2381. 16783025 10.1534/genetics.105.052506 PMC1569719

[42] Lawson HA, Zelle KM, Fawcett GL, Wang B, Pletscher LS, et al (2010) Genetic, epigenetic, and gene-by-diet interaction effects underlie variation in serum lipids in a LG/J×SM/J murine model. J Lipid Res 51: 2976–2984. 20601649 10.1194/jlr.M006957 PMC2936764

[43] Gagne S, Dehbi L, Lequere D, Cayer F, Morin JL, et al (1993) Increase of greenhouse tomato fruit yields by Plant Growth- Promoting Rhizobacteria (PGPR) inoculated into the peat-based growing media. Soil Biol Biochem 25: 269–272.

[44] Wurst S, Dugassa-Gobena D, Langel R, Bonkowski M, Scheu S (2004) Combined effects of earthworms and vesicular-arbuscular mycorrhizas on plant and aphid performance. New Phytol 163: 169–176. 10.1111/j.1469-8137.2004.01106.x 33873788

[45] Dodd IC, Zinovkina NY, Safronova VI, Belimov AA (2010) Rhizobacterial mediation of plant hormone status. Ann Appl Biol 157: 361–379.

[46] Pate J, Arthur D (1998) δ13C analysis of phloem sap carbon: novel means of evaluating seasonal water stress and interpreting carbon isotope signatures of foliage and trunk wood of *Eucalyptus globulus* . Oecologia 117: 301–311. 28307909 10.1007/s004420050663

[47] Hale BK, Bale JS, Pritchard J, Masters GJ, Brown VK (2003) Effects of host plant drought stress on the performance of the bird cherry-oat aphid, *Rhopalosiphum padi* (L.): a mechanistic analysis. Ecol Entomol 28: 666–677.

[48] Pieterse CMJ, Dicke M (2007) Plant interactions with microbes and insects: from molecular mechanisms to ecology. Trends Plant Sci 12: 564–569. 17997347 10.1016/j.tplants.2007.09.004

[49] Li AL, Meng CS, Zhou RH, Ma ZY, Jia JZ (2006) Assessment of Lipid Transfer Protein (LTP1) Gene in Wheat Powdery Mildew Resistance. Agricultural Sciences in China 5: 241–249.

[50] Steffenson BJ, Hayes P, Kleinhofs A (1996) Genetics of seedling and adult plant resistance to net blotch (*Pyrenophora teres* f. *teres*) and spot blotch (*Cochliobolus sativus*) in barley. Theor Appl Genet 92: 552–558. 24166322 10.1007/BF00224557

[51] Zhong S, Effertz RJ, Jin Y, Franckowiak JD, Steffenson BJ (2003) Molecular mapping of the leaf rust resistance gene *Rph6* in barley and Its linkage relationships with *Rph5* and *Rph7* . Phytopathology 93: 604–609. 18942983 10.1094/PHYTO.2003.93.5.604

[52] Underwood N, Rausher MD (2000) The effects of host-plant genotype on herbivore population dynamics. Ecology 81: 1565–1576.

[53] Bonkowski M, Geoghegan IE, Birch ANE, Griffiths BS (2001) Effects of soil decomposer invertebrates (protozoa and earthworms) on an above-ground phytophagous insect (cereal aphid) mediated through changes in the host plant. Oikos 95: 441–450.

[54] Wooley SC, Paine TD (2007) Can intra-specific genetic variation in arbuscular mycorrhizal fungi (*Glomus etunicatum*) affect a mesophyll-feeding herbivore (*Tupiocoris notatus* Distant)? Ecol Entomol 32: 428–434.

[55] Poveda K, Steffan-Dewenter I, Scheu S, Tscharntke T (2005) Effects of decomposers and herbivores on plant performance and aboveground plant-insect interactions. Oikos 108: 503–510.

[56] Hamilton EW, Frank DA (2001) Can plants stimulate soil microbes and their own nutrient supply? Evidence from a grazing tolerant grass. Ecology 82: 2397–2402.

[57] Gatehouse JA (2002) Plant resistance towards insect herbivores: a dynamic interaction. New Phytol 156: 145–169. 10.1046/j.1469-8137.2002.00519.x 33873279

[58] Li XC, Schuler MA, Berenbaum MR (2002) Jasmonate and salicylate induce expression of herbivore cytochrome P450 genes. Nature 419: 712–715. 12384696 10.1038/nature01003

[59] Broadway RM (1997) Dietary regulation of serine proteinases that are resistant to serine proteinase inhibitors. J Insect Physiol 43: 855–874. 12770497 10.1016/s0022-1910(97)00028-0

